# Medical Secrets

**Published:** 1992-03

**Authors:** J. D. Davies

**Affiliations:** University of Bristol


### Book Reviews

**MEDICAL SECRETS**
**A. J. Zollo, Philadelphia**
Hanley & Belfus, 1991. 558 pp. + xiii. Price: not stated.
ISBN 1 56053 011 1

Michael Wilson passed this book for review to me. The truth of the matter is that it should have been sent on to a proper front-line clinician. However, it has rather got a grip on its recipient. What sorts of fish poisoning do you know? Have you even heard of Poncet's disease?

The awful thing is that all these fascinating byways of knowledge now seen to be expected of MCQ or viva candidates for examinations. Hence, the sales pitch of the blurb coming with the book, which says that it contains questions which you will be asked on ward rounds, in the clinic, and in oral examinations.

All that said, this is still a book for dipping into. The American English is easily readable, and the tables are helpful. None the less, it defies the imagination to think that trainees in any clinical discipline should have to know that on chromosome 9 there are "genes in the breakpoint cluster region (bcr) of 22 cell lines established from CML expressing a new messenger RNA which reflects the chimeric gene produced by the fusion of the bcr and c-able genes".

Don't let these random comments put you off. Here is a book framed in the new language of coded MCQ'ese. It is attractively produced in paperback, and well worth reading; that is if you are in the happy position of not having to sit an examination next week.

J. D. Davies

University of Bristol

